# Dabigatran level monitoring prior to idarucizumab administration in patients requiring emergent cardiac surgery

**DOI:** 10.1007/s11239-017-1587-9

**Published:** 2017-12-08

**Authors:** Radoslaw Litwinowicz, Janusz Konstanty-Kalandyk, Tadeusz Goralczyk, Krzysztof Bartus, Piotr Mazur

**Affiliations:** 1 Department of Cardiovascular Surgery and Transplantology, The John Paul II Hospital, Kraków, Poland; 2 Institute of Cardiology, Jagiellonian University Medical College, 80 Prądnicka St., Kraków, 31-202 Poland; 3 Central Laboratory, The John Paul II Hospital, Kraków, Poland

To the Editor,

Non-vitamin-K antagonist oral anticoagulants (NOACs), including dabigatran (a direct factor IIa [FIIa] inhibitor) increasingly replace the vitamin K antagonists (VKAs) for favorable risk–benefit profile [1] and lower risk of major bleeding [2] in atrial fibrillation. NOACs also significantly reduce the risk of recurrent venous thromboembolism (VTE), and compared with VKAs present lower risk of bleeding in this group [3]. The use of NOACs increases in VTE prevention, even though in the setting of surgical emergency or life-theratening bleeding, the NOAC therapy may be dangerous [4].

In 2015, the FDA approved idarucizumab for dabigatran reversal in emergency situations [5]. Idarucizumab is a monoclonal antibody fragment that binds dabigatran with high affinity, and presents good clinical outcomes [5, 6]. Current European Heart Rhythm Association (EHRA) practical guidelines recommend idarucizumab for life-threatening bleeding, or prior to emergency surgery in dabigatran treated patients [7].

Clinical experience with idarucizumab in cardiac surgery is currently limited. In our institution, we managed several dabigatran-treated patients in emergency cardiosurgical setting [8]. In previous cases, the clinical decision to administer idarucizumab was made following emergency laboratory assessment of baseline dabigatran level (both individuals required an open-heart surgery for acute aortic syndrome) [8]. However, in specific clinical scenarios, monitoring of dabigatran level may be challenging and potentially impede the decision to use the expensive idarucizumab preparation based just on uncertain dabigatran intake history, and exposing the patient to the risk of excessive (and potentially lethal) surgical bleeding, if dabigatran intake history is uncertain.

We report a case of a 63-years-old patient who received dabigatran for VTE and required emergency coronary artery bypass grafting (CABG) for an acute coronary syndrome (ACS) with coronary anatomy precluding percutaneous coronary intervention (PCI), in whom the preoperative dabigatran level measurement was futile because of interferences with other thrombin inhibitors.

## Case presentation

A 63-years-old male patient with a history of stable coronary artery disease (CAD), previous myocardial infraction (MI), deep venous thrombosis, polycythemia vera and arterial hypertension was admitted to our Institution (tertiary cardiac care center) for the surgical management of a new-onset ACS in form of ST-segment elevation MI. He received dabigatran due to VTE (2 × 150 mg/day; last dose intake on the day of surgery) [3]. The patient was admitted to a local hospital due to severe chest pain. As soon as the ACS diagnosis was made, the coronary angiography was performed, showing a multi-vessel coronary disease with critical stenosis (99%) of three arteries, and impending occlusion of the critically stenotic left main coronary artery (the right coronary artery was recessive). Because of the unfavorable anatomy, the patient was consulted online with the emergency Heart Team, started on unfractionated heparin (UFH) in continuous infusion, and transferred to our department for emergency CABG.

The surgical Team was called in, and dabigatran level, thrombin time, reptilase time, activated partial thromboplastin time (APTT) and prothrombin time were measured on admission. His renal function was preserved (estimated glomerular filtration rate was 75 ml/min).

Dabigatran plasma concentration was determined on the BCS-XP automated analyzer (Siemens Healthcare Diagnostics Products GmbH, Marburg, Germany), using the Hemoclot thrombin inhibitors (HTI) assay (Hyphen BioMed, Neuville-Sur-Oise, France). The assay based on modified diluted thrombin time was calibrated with the calibrators (Biophen^®^ Dabigatran Calibrator Low) containing different concentrations of dabigatran (0, 56 and 108 ng/mL). A set of two levels control plasmas of dabigatran (Biophen^®^ Dabigatran Control Low) was used for the quality control of measurements. Reproducibility at dabigatran concentrations of 29 and 80 ng/mL were 12.7 (n = 10) and 8.7% (n = 10), respectively.

Unfortunately, the measurement of dabigatran plasma concentration was non-diagnostic because of the continuous UFH infusion. Nevertheless, the laboratory test was repeated five times in different time intervals. All laboratory results are present in Table 1. In spite of the inability to measure the dabigatran concentration, the patient received 5 g of intravenous idarucizumab in two 50-ml bolus infusions (each containing 2.5 g of idarucizumab, no more than 15 min apart, directly before the operation).

**Table 1:** Laboratory findings in patient with ACS and receiving dabigatran treatment with continuous heparin infusion

	Measurement				
	I (17:30)	II (18:20)	III (21:40)	IV (22:30)	V (24:00)
Time period	Baseline	50 min	4 h 10 min	5 h 00 min	6 h 30 min
Patient clinical status	Admission to the hospital, continuous heparin infusion	Preoperative department, continuous heparin infusion	Just before surgery, directly before idarucizumab administration	Cardiac surgery- CPB, full dose heparin therapy	After CPB, heparin reversal using protamine
TT (s) [16.0–21.0]	81.3	> 150	25.2	> 150	25.5
RT (s) [16.0–22.0]	14.2	14	13.2	13	14.2
APTT (s) [25.9–36.6]	151.6	> 300	57.1	> 300	48.3
PT (s) [10.4–13.0]	15.3	> 170	14.4	> 170	17.6
DABIGATRAN level (ng/mL)	37	ND	33	ND	57

*CPB* Cardio-pulmonary bypass, *ND* not done, *PT* prothrombin time, *RT* reptilase time, *APTT* activated partial thromboplastin time, *TT* thrombin time

After idarucizumab administration, CABG in normothermic cardiopulmonary bypass (CPB) followed. The saphenous vein grafts were placed to marginal and diagonal coronary arteries, and the left internal mammary artery was grafted to the left anterior descending coronary artery, following the standardized procedures. CPB time was 97 min, aortic cross clamp was 52 min, the overall procedural time was 140 min. Despite the scrupulous surgical hemostasis, the postoperative bleeding, according to the universal definition of perioperative bleeding in cardiac surgery, was severe [9]. The chest tube output was 840 ml after 24 h, and a total of two units of red blood cells, four units of platelets and eight units of fresh frozen plasma were administered in the first 24 h after the procedure.

During the postoperative course, the patient required antibiotics for postoperative pneumonia, diuretic treatment and intensive pulmonary rehabilitation. Antiplatelet regimen with aspirin (150 mg/day) was reinitiated on postoperative day 1. Dabigatran (2 × 150 mg) was restarted on postoperative day 3. On postoperative day 10, the patient was discharged to a local rehabilitation facility.

## Discussion

In contrast to vitamin K antagonists, dabigatran is reported to have a shorter half-life and thus narrower therapeutic window. In patients with normal renal function, the restoration of hemostasis is expected within 12–24 h after last dose intake [10]. Surgery or intervention should ideally be deferred, until 24 h after the last dose [10]. However, the real-life clinical practice shows that there are many situations, where reversal of dabigatran is required immediately. RE-VERSE AD trial demonstrated that idarucizumab is efficacious in dabigatran reversal [5]. Of note, in the RE-VERSE AD trial, in each case dabigatran and idarucizumab plasma levels were measured to assess the dabigatran reversal effect [5, 6].

The American Heart Association Scientific Statement from 2017 suggests for major bleeding in the event of dabigatran intake in emergency cases: compression when possible, supportive measures, and upfront idarucizumab [11]. Following the RE-VERSE AD trial, also in real-life cases dabigatran laboratory monitoring is performed before idarucizumab administration [8, 12, 13].

However, in expert opinions, in life-threatening situations, when the last dose of dabigatran was intake < 12 h or there is uncertainty about the timing of last ingestion, it may be necessary to consider idarucizumab before the dabigatran level the results are known [12, 14]. Still, there are no specific recommendations for dabigatran level monitoring after idarucizumab administration.

Our patient, who was taking dabigatran for VTE prophylaxis, required a life-saving CABG, and in this specific clinical scenario the continuous preoperative infusion of UFH was mandatory [15]. Both anticoagulants (dabigatran and heparin) inhibit the same level of coagulation cascade: dabigatran is a direct thrombin inhibitor, while heparin inhibits thrombin indirectly [16]. Therefore, because dabigatran and heparin have similar effects on thrombin, the coagulation tests used were clinically not useful for decision making.

The HTI method is based on the measurement of the diluted tested plasma clotting time after addition of the constant amount of human thrombin. Prolonged clotting times are associated with higher dabigatran concentrations. However, the HTI assay does not contain any heparin inhibitors, which is why heparin (or other thrombin inhibitors) present in the tested sample may interfere with the assay, and prolong the clotting time, producing falsely high plasma concentrations of dabigatran.

Normal reptilase time, and at the same time prolonged thrombin time, indicate that heparin is present in all tested samples. Thus, one might speculate that the dabigatran concentrations in samples I, III and V are falsely increased, however it is difficult to estimate how much. The thrombin time, prothrombin time and APTT measurement above the normal reference range in samples II and IV suggest that these samples contained heparin at very high concentrations. It was impossible to measure the concentration of dabigatran in these samples using the HTI.

Fast but reliable measurement of dabigatran concentrations in patients concomitantly receiving heparin is a challenge. The gold standard in accurate dabigatran concentrations measurement is liquid chromatography/tandem mass spectrometry (LC–MS/MS) [17], however, the availability of this method is limited, especially in the emergency setting. The HTI belongs to the group of commercially available “specific coagulation tests”, which is favorable to LC–MS/MS in some aspects, though susceptible to interference by heparin [18].

The new commercially available test for dabigatran quantification irrespectively of heparin intake is the chromogenic Innovance^®^ DTI Assay (Siemens Healthcare Diagnostics Products GmbH, Marburg, Germany). According to the manufacturer, dabigatran measurement assays are not influenced by heparin concentrations of 8 and 15 IU/mL, respectively. Another test based on diluted thrombin time Hemosil Direct Thrombin Inhibitor (Instrumentation Laboratory) is insensitive to UFH up to 2.2 IU/mL. In any case, care must be taken during sampling, to avoid additional contamination of test tubes with heparin.

Our case showed, that there are emergency situations, where laboratory measurement of dabigatran level may not be clinically useful because of drugs interactions that mask the anticoagulant effect of dabigatran. In such cases, HTI assay may be unrevealing. In life-threatening situations, when urgent surgery/procedure cannot be delayed, and if the patient received dabigatran within < 12 h (or when the timing of last dose intake is uncertain), idarucizumab should be administered without dabigatran level monitoring.

There is also a question why we observed an excessive postoperative bleeding despite idarucizumab administration. In previous cases the observed bleeding was moderate, however it might be speculated that the longer procedural time was a reason to transfuse the patient intraoperatively, while in the current case, with shorter procedural time, the transfusions followed in the intensive care unit [5]. Furthermore, there is evidence that plasma dabigatran levels may increase by up to 96% of the baseline value after successful initial neutralization by idarucizumab due to redistribution, and another idarucizumab dose is required [12]. Because of nondiagnostic measurement, we cannot exclude this phenomenon in our patient.

In conclusion, we report that dabigatran level monitoring may not always be feasible in patients requiring idarucizumab before an emergency cardiac surgery if UFH infusion is started. In our case, due to common affinity of dabigatran and heparin to thrombin, objective measurement of dabigatran could not be performed with the available test. We conclude that in a patient with any suspicion of recent dabigatran intake, in whom the drug level cannot be measured and the surgery deferred, idarucizumab should be administered in any case. Heparin insensitive dabigatran measurements should be preferred in cardiac surgery centers.
